# Liver cirrhosis mortality, alcohol consumption and tobacco consumption over a 62 year period in a high alcohol consumption country: a trend analysis

**DOI:** 10.1186/s13104-015-1808-2

**Published:** 2015-12-26

**Authors:** Ulrich John, Monika Hanke

**Affiliations:** Institute of Social Medicine and Prevention, University Medicine Greifswald, Walther-Rathenau-Str. 48, 17475 Greifswald, Germany

**Keywords:** Liver cirrhosis mortality, Alcohol consumption, Tobacco smoking, School education, Trends

## Abstract

**Background:**

The relationship between alcohol consumption and liver cirrhosis mortality has been revealed by data from several different countries. However, the impact of tobacco smoking on liver cirrhosis has not been considered. The aim of this study was to estimate trends in liver cirrhosis mortality and alcohol and tobacco consumption from 1952 to 2013 as well as more recent trends in substance use disorder treatments and hospital treatments of liver diseases in Germany.

**Methods:**

Data from the National Statistics Office were used. Liver cirrhosis was diagnosed according to the International Classification of Diseases (ICD-6 to ICD-10). Alcohol beverages and tobacco products were estimated according to tax or governmental data. Substance use disorder treatment and hospital treatment data were used. Trends were calculated using Joinpoint regression analyses.

**Results:**

Liver cirrhosis mortality among men increased annually by 8.4 % from 1952 to 1960 and increased annually by 2.8 % from 1961 to 1976. From 1976 to 1982, liver cirrhosis mortality decreased annually by 4.8 %, from 1982 to 2013 liver cirrhosis mortality decreased annually by 1.2 %. Among females, liver cirrhosis mortality increased annually by 8.9 % from 1952 to 1959 and by 4.3 % from 1959 to 1968, but then decreased 1.0 % annually from 1968 to 1995. After 1995, liver cirrhosis mortality decreased 1.9 % annually through 2013. These reductions in liver cirrhosis mortality were accompanied by decreases in alcohol consumption beginning in 1976. These findings were also accompanied by decreases in the consumption of cigarette equivalents since 1971. Meanwhile, the number of substance use disorder treatments and hospital treatments of liver diseases increased.

**Conclusions:**

The decrease in liver cirrhosis mortality may have been caused by a decrease in alcohol drinking and tobacco smoking. Smoking may have exerted indirect effects via alcohol consumption as well as direct effects. These trends existed despite largely missing preventive efforts to reduce alcohol consumption and tobacco smoking. Increases in educational attainment in the general population may have contributed to the reductions in alcohol and tobacco consumption. Convincing evidence that the increased provision of substance use disorder treatment significantly contributed to the decrease of liver cirrhosis was not found.

**Electronic supplementary material:**

The online version of this article (doi:10.1186/s13104-015-1808-2) contains supplementary material, which is available to authorized users.

## Background

Worldwide, liver cirrhosis mortality has decreased since the mid-1970s [1]. For European countries, liver cirrhosis mortality increased from 1959 until the 1970s but was subsequently followed by a decrease in liver cirrhosis mortality [2]. In the United States, similar trends have been found [3].

The relationship between liver cirrhosis mortality and alcohol consumption has been identified in data collected from several countries [4, 5], including decreases in liver cirrhosis mortality after preventive efforts to reduce alcohol consumption [6]. Dose–response relationships between alcohol consumption and liver cirrhosis mortality also exist [7]. Latencies in the time between changes in alcohol consumption and rates of liver cirrhosis mortality of less than 1 year have been found [8]. Potential reasons for decreases in liver cirrhosis mortality can be analyzed using a time trends study design utilizing alcohol tax data. This approach has the advantage of introducing only a small amount of bias compared with estimates of alcohol consumption using national survey data. These surveys, estimated using alcohol production data, have been shown to disclose just 40–60 % of true alcohol consumption [9].

One limitation of time-trend analysis studies is that previously tobacco smoking had not been considered as having an impact on liver cirrhosis through alcohol consumption. Because alcohol consumption and smoking have been found to be interrelated [10] smoking might contribute to sustaining a high level of alcohol consumption, thereby indirectly increasing the risk of liver cirrhosis. Additionally, direct relationships between smoking and liver cirrhosis exist. In a population cohort study, among those who smoked 11 or more grams of tobacco per day, women had a hazard ratio of 2.2 (1.4–3.4) for liver cirrhosis and men had a hazard ratio of 1.4 (0.9–2.2) for liver cirrhosis compared to female and male never smokers after adjustment for alcohol consumption, respectively [11]. Twelve percent of liver cirrhosis cases among women and 6 % of liver cirrhosis cases among men have been attributed to smoking [11]. In a sample of 1,290,413 women smoking was predictive for liver disease after 6 years [12]. Despite drinking less than 50 grams of pure alcohol per week, women had a relative risk of 2.9 (2.4–3.5) for liver cirrhosis if they were current smokers [12]. Female smokers who drank 50 grams or more of pure alcohol per week, had a relative risk for liver cirrhosis of 8.0 (6.8–9.4) compared to female non-smokers who drank less than 50 grams of pure alcohol per week. There was a dose–response relationship among women who consumed 50 g or more of pure alcohol per week: the more the women smoked, the higher their relative risk of liver cirrhosis [12].

The aim of this paper is to first analyze trends of liver cirrhosis mortality and alcohol consumption in Germany over a 62 year period through the year 2013. Second, trends in tobacco use will be analyzed, and a correspondence of tobacco use with liver cirrhosis mortality will be estimated. Third, more recent trends of the number of substance use disorder (SUD) treatments provided and hospital treatments for alcohol-related liver disease will be analyzed.

## Methods

### Data sources

Annual data concerning liver cirrhosis mortality, amounts of alcohol and taxed tobacco products, were provided for each year from 1952 to 2013, data about treatments of SUD for 1982–2013, and data about hospital treatments of liver diseases for 2000–2013 by the National Statistics Office for the Federal Republic of Germany. Until 1990 the data were collected from West Germany only, from 1991 to 2013 the data were collected from all of Germany. Because of the German reunification no data for the year 1990 were available. We used the mean of 1989 and 1991 for the year 1990. All data are standardized to Germans based on the national population of those who were of drinking and smoking age (15 or older). If possible data were stratified by sex.

For the estimation of deaths from liver cirrhosis, we used the causes of death statistics from the Federal Republic of Germany. Amounts of alcohol were provided by tax or governmental data as liters of beer or wine produced minus exports, plus imports (only exception: beer from countries outside the European Union). The proportion of pure alcohol for beer was calculated as 4.8 % alcohol per volume. Non-alcoholic beer from 1993 on was excluded from the tax statistics. The proportion of pure alcohol for wine or sparkling wine was calculated as 11.0 % alcohol per volume [13]. The amount of pure alcohol in spirits was provided by the producers via tax authorities to the National Statistics Office. Tobacco tax statistics from the National Statistics Office provided the number of cigarettes, number of cigars or small cigars, tons of fine-cut tobacco, and tons of pipe tobacco for each year between 1952 and 2013 [19, 20]. For the calculation of the number of SUD treatments data were provided by the institution that finances these treatments (German Pension Insurance). SUD included alcohol dependence and drug dependence. In the years 2003–2013, 67–74 % of the male and 76–81 % of the female patients were treated for alcohol dependence [14]. The number of SUD treatments in Germany has been documented since 1982. We used the data for each year from 1982 to 2013. Hospital inpatient diagnoses at discharge were provided according to the International Classification of Diseases (ICD; codes with 3 digits).

### Outcomes

For liver cirrhosis mortality the diagnosis “liver cirrhosis” was identified according to ICD-6, code 581, for the years 1952–1957, according to ICD-7, code 581, for the years 1958–1967, according to ICD-8, code 571, for the years 1968–1978, according to ICD-9, code 571.2, alcoholic liver cirrhosis, and 571.5, liver cirrhosis with alcohol not specified, for the years 1979–1997, and according to ICD-10, code K70.3, alcohol liver cirrhosis, and K74.6, other or unspecified liver cirrhosis, for the years 1998–2013.

Alcohol consumption was estimated as the amount of pure alcohol divided by the population at age 15 or older for each year [9]. Cigarette equivalents were used to estimate tobacco smoked per year. Number of cigars or small cigars, tons of fine-cut tobacco and tons of pipe tobacco were converted to cigarette equivalents using one gram of fine-cut or pipe tobacco as one cigarette equivalent and one cigar or small cigar as two cigarette equivalents according to standard conventions of the Organization for Economic Co-operation and Development [21]. For SUD treatments, we used the number of SUD treatments given per year. This treatment is provided as both an inpatient or outpatient service, with the aim of supporting patients in achieving long-term abstinence. For hospital treatments of liver diseases inpatient diagnoses we used the ICD-10-diagnosis groups K70 and K74 from 2000 onwards. K70 includes alcoholic liver diseases: fatty liver, hepatitis, fibrosis and sclerosis, cirrhosis, and liver failure. K74 includes non-alcohol-related fibrosis and cirrhosis of the liver.

### Ethical considerations

The study adheres to the STROBE statement (http://www.strobe-statement.org).

### Statistical analysis

Trends were calculated using Joinpoint regression analyses according to the program Joinpoint 4.1.1.3 [15]. It provides begin and end of single trends. Annual percent changes as a measure of the strength of a trend are reported. Insignificant annual percent changes were interpreted as stable. For liver cirrhosis mortality, alcohol, and cigarette equivalents, we defined a maximum number of four trends. At a maximum, two trends were allowed both for SUD treatments and hospital treatments, due to the few number of years available for analysis.

### Availability of supporting data

All data used in the Joinpoint analyses are provided in Additional files 1, 2 and 3.

## Results

**Fig. 1:**
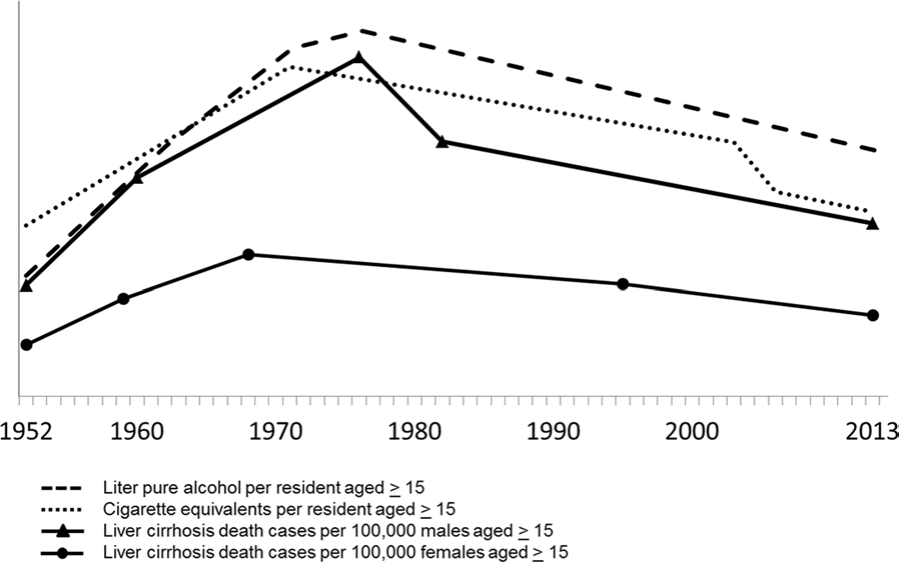
Trends of liver cirrhosis mortality, alcohol and tobacco sales and alcohol treatment

Liver cirrhosis mortality increased from 1952 to 51.4 per 100,000 men and 21.4 per 100,000 women aged 15 or older in 1976. In 2013 liver cirrhosis mortality was 26.2 per 100,000 men and 12.3 per 100,000 women aged 15 or older. Among men, trend analyses revealed that liver cirrhosis mortality increased annually by 8.4 % from 1952 to 1960 and annually by 2.8 % from 1960 to 1976. From 1976 to 1982 liver cirrhosis mortality decreased annually by 4.8 %, after 1982 liver cirrhosis mortality decreased annually by 1.2 % until 2013 (Table 1; Fig. 1). Among females, liver cirrhosis mortality increased annually by 8.9 % from 1952 to 1959 and by 4.3 % annually from 1959 to 1968. Liver cirrhosis mortality then decreased by 1.0 % annually until the year 1995, at which point liver cirrhosis mortality decreased by 1.9 % annually until 2013. The ratio of liver cirrhosis mortality for men to women remained constant from 1952 to 2013 and ranged from 2.1 to 2.3.

**Table 1:** Trends in liver cirrhosis mortality, estimated alcohol consumption, cigarette smoking, specialized alcohol treatment and hospitalization for liver disease

	Trend no.												Trend		
	1			2			3			4			Entire time span		
	Years	APC	CI	Years	APC	CI	Years	APC	CI	Years	APC	CI	Years	AAPC	CI
Liver cirrhosis mortality															
Males	1952–1960	+8.4	+6.8 to +10.0	1960–1976	+2.8	+2.2 to +3.4	1976–1982	−4.8	−7.6 to −1.8	1982–2013	−1.2	−1.4 to −1.0	1952–2013	+0.7	+0.3 to +1.1
Females	1952–1959	+8.9	+7.1 to +10.8	1959–1968	+4.3	+2.9 to +5.8	1968–1995	−1.0	−1.3 to −0.8	1995–2013	−1.9	−2.3 to −1.5	1952–2013	+0.6	+0.3 to +0.9
Alcohol liters															
	1952–1962	+7.9	+7.5 to +8.4	1962–1971	+3.4	+2.8 to +4.0	1971–1976	+0.7 ns	−0.9 to +2.4	1976–2013	−1.0	−1.1 to −1.0	1952–2013	+1.2	+1.0 to +1.4
Cigarette equivalents															
	1952–1971	+3.6	+3.3 to +3.9	1971–2003	−0.9	−1.1 to −0.8	2003–2006	−6.7 ns	−15.7 to +3.3	2006–2013	−1.2 ns	−2.5 to +0.2	1952–2013	+0.1 ns	−0.4 to +0.7
Substance use disorder treatments															
	1982–2009	+3.1	+2.7 to +3.6	2009–2013	−2.9 ns	−9.8 to +4.5							1982–2013	+2.3	+1.3 to +3.3
Hospital treatments ICD10: K70															
Males	2000–2013	+2.4	+1.7 to +3.0										2000–2013	+2.4	+1.7 to +3.0
Females	2000–2013	+1.7	+1.1 to +2.2										2000–2013	+1.7	+1.1 to +2.2
Hospital treatments ICD10: K74															
Males	2000–2009	−3.2	−5.0 to −1.4	2009–2013	+3.4 ns	−3.2 to +10.4							2000–2013	−1.2 ns	−3.3 to +0.8
Females	2000–2009	−1.9	−2.9 to −0.9	2009–2013	+3.4 ns	−0.1 to +7.0							2000–2013	−0.3 ns	−1.4 to +0.8
Hospital treatments ICD10: K70 or K74															
Males	2000–2013	+1.0	+0.7 to +1.2										2000–2013	+1.0	+0.7 to +1.2
Females	2000–2013	+0.5	+0.3 to +0.8										2000–2013	+0.5	+0.3 to +0.8

Liver cirrhosis mortality: death cases per 100,000 male/female population aged 15 or older

Alcohol liters: per resident aged 15 or older

Cigarette equivalents: per resident aged 15 or older

Substance use disorder treatments: per 100,000 residents aged 15 or older, 2 trends at maximum allowed

Hospital treatments: inpatient treatments, discharge diagnosis group ICD-10, K70 or K74, per 100,000 residents aged 15 or older, 2 trends at maximum allowed

ICD-10: K70 Alcoholic liver diseases

ICD-10: K74 Non-alcohol-related fibrosis or cirrhosis of the liver

*APC* annual percent change, *CI* 95 %-confidence interval, *ns* not significant, *AAPC* average annual percent change

Alcohol consumption increased until 1976 but then decreased from 1,661,924 liters per 100,000 population aged 15 or older in 1976 to 1,391,211 liters in 1986 and 1,112,716 liters in 2013. Trend analysis revealed that alcohol consumption from 1952 to 1962 increased annually by 7.9 percentage points; from 1962 to 1971, alcohol consumption increased annually by 3.4 %. In 1971 consumption leveled off until 1976, and beginning in 1976 consumption decreased annually by 1.0 % until 2013.

The number of cigarette equivalents per 100,000 population aged 15 or older was 292 million in 1971 and 163 million in 2013. The consumption of cigarette equivalents increased annually by 3.6 % from 1952 to 1971, and after 1971 decreased annually by 0.9 % until 2003. After 2003, cigarette equivalents consumption remained unchanged until 2013.

The number of SUD treatments increased from 32.7 per 100,000 in 1982 to 81.2 per 100,000 population aged 15 or older in 2009. This corresponds to an annual increase of 3.1 %. After 2009, the number of SUD treatments remained constant until 2013. From 2000 to 2013, hospital treatments of alcohol-related liver diseases (ICD-10, K70) increased among men by 2.4 % annually and among women by 1.7 % annually. With regards to non-alcoholic liver diseases (ICD-10, K74), after a decrease from 2000 to 2009 (males: −3.2 % annually, females: −1.9 % annually) numbers remained unchanged from 2009 to 2013.

## Discussion

Three main results were found: first, liver cirrhosis mortality decreased starting in 1977 among males and in 1969 among females. This trend was accompanied by decreases in alcohol consumption beginning in 1977. Second, these results were also accompanied by decreases in consumption of cigarette equivalents starting in 1972. Third, liver cirrhosis mortality decreased whereas the number of SUD treatments and hospital treatments for alcohol-related liver disease increased.

The reduction in liver cirrhosis mortality is similar to trends found in other European countries. Data from other countries have also revealed a decrease in liver cirrhosis mortality since the mid-1970s [2]. This trend has been confirmed in additional countries outside of Europe [1] including the United States [3]. This alignment suggests two potential causes for the reduction in liver cirrhosis mortality: a decrease in alcohol consumption and improvement in treatments.

The decrease in pure alcohol consumption over the course of 37 years from 16.62 liters consumed annually per resident aged 15 or older to 11.13 liters consumed annually is accompanied by a decrease in liver cirrhosis mortality of approximately half: from 51.4 per 100,000 male residents aged 15 or older in 1976 to 26.2 in 2013 and from 21.4 per 100,000 female residents aged 15 or older in 1976 to 12.3 in 2013. Earlier evidence showed simultaneous changes in the relationship between alcohol consumption and liver cirrhosis mortality in a dose–response relationship pattern [7].

Potential reasons for the decrease in alcohol consumption might include public health activities aiming to reduce alcohol consumption in the general population, further changes in social norms with regards to alcohol consumption, changes in the economy, mechanisms of the beverage market, changes in educational attainment, and reductions in tobacco consumption. Public health activities designed to reduce alcohol consumption did not take place in a way that might be strong enough to make changes. However, drinking norms may have changed over time, e.g., the acceptability of drinking and driving. Alcohol-free driving was advertised publicly. Additionally, media covering other countries with strict alcohol policies may have had an influence. General health-related norms may also have become more influential in reducing alcohol consumption. An economic recession in 1967 may have added to the decrease in alcohol consumption during this time period [cf. 16, 17]. The increase in the proportion of people attaining the highest level of education may have contributed to improved health consciousness and decreased alcohol consumption. The rate of those with 12 or more years of education among the female population at age 19 was 3.1 % in 1950 and 37.7 % in 2013; among men, the rate of those with 12 or more years of education was 6.1 % in 1950 and 29.8 % in 2013 [18, 19].

The finding that the decline in smoking started 5 years earlier than alcohol consumption and liver cirrhosis mortality suggests an indirect and direct effect of smoking on the decrease in liver cirrhosis mortality may have been active. The presence of indirect effects is supported by evidence concerning alcohol drinking and tobacco smoking [10, 20]. The probability of alcohol dependence increases as more cigarettes per day are smoked [21]. If tobacco smoking becomes less prevalent, the “appetite” for alcohol may decrease [21, 22]. A direct link between tobacco smoking and liver cirrhosis is also suggested by evidence. Cohort data revealed that tobacco smoking may increase the risk of liver cirrhosis independent of the influence of alcohol [11, 12, 23, 24]. Even among those who consumed less than 7 drinks per week, female current smokers had a higher risk for liver cirrhosis than non-smokers [12]. Animal studies suggest independent associations between tobacco smoking and liver cirrhosis [10, 25].

Improvements in SUD treatments might be an additional reason for the decrease in liver cirrhosis over time. In Germany, the number of facilities for treating alcohol dependence increased steadily since 1982. However, data on the adult general population revealed that only 22.8 % of alcohol dependent subjects utilized this treatment [26]. Among those who had taken part in specialized alcohol treatment, no additional subjects had survived 14 years after the baseline interview than among those who did not utilize specialized alcohol treatment services [26]. These findings do not speak in favor of SUD treatment as contributing significantly to the decrease in liver cirrhosis.

The increase in hospital treatments for alcohol-related liver disease since 2000 might be of some relevance for explaining the decrease in liver cirrhosis mortality. One reason for the increase in hospital treatments might be increased detection rates in primary medical care [27]. Fewer individuals may have gotten to a stage of severe impairment, a stage in which cirrhosis develops. Treatments and secondary prevention of liver disease have also likely improved [28] as have the survival rates of patients with liver cirrhosis [29, 30]. However, data from the UK revealed no progress in survival rates during almost five decades through 1999 [31, 32].

The strengths of the present study include the time span of 62 years covered by the data for liver cirrhosis mortality and alcohol and tobacco sales and the data being largely free of bias except the diagnosis of liver cirrhosis as the cause of death. Limitations include that liver cirrhosis may have been underestimated. For example, cases of death that occurred outside the hospital may not have been captured. Data from a previous study revealed that less than half of liver cirrhosis deaths detected by autopsy had been declared in mortality statistics [33]. Additionally, the proportion of alcohol-related liver cirrhosis among all liver cirrhosis cases may have been reduced over time. Changes in liver cirrhosis rates resulting from the obesity epidemic may increase the proportion of non-alcoholic fatty liver disease [34].

## Conclusions

Liver cirrhosis mortality among males and alcohol and tobacco consumption started to decline within 5 years of one another. The data support that in addition to reduced alcohol consumption reductions in tobacco smoking may have added to decreases in liver cirrhosis mortality over time. Although the number of specialized treatments for SUD increased during this time period as well, we could not confirm that it significantly contributed to the decrease in liver cirrhosis mortality.

## Additional files

10.1186/s13104-015-1808-2 Data file 1 (alcohol liters, cigarette equivalents, liver cirrhosis death cases).
10.1186/s13104-015-1808-2 Data file 2 (substance use disorder treatments).
10.1186/s13104-015-1808-2 Data file 3 (hospital treatment cases).
