# Supplementary material for: Liver cirrhosis mortality, alcohol consumption and tobacco consumption over a 62 year period in a high alcohol consumption country: a trend analysis
Source: BMC Res Notes. 2015 Dec 26;8:822. doi: 10.1186/s13104-015-1808-2 (PMC4691532; doi:10.1186/s13104-015-1808-2)
Supplement: Supplementary file 1 — 10.1186/s13104-015-1808-2 Data file 1 (alcohol liters, cigarette equivalents, liver cirrhosis death cases). [file 13104_2015_1808_MOESM1_ESM.rtf]

Data file 1 alcl  		alcohol litres per resident aged 15 or oldercig   		cigarette equivalents per resident aged 15 or olderlcdcm 	liver cirrhosis death cases aged 15 or older, menlcdcf 		liver cirrhosis death cases aged 15 or older, womenlcmom  	liver cirrhosis death cases per 100,000 male population aged 15 or olderlcmof  	liver cirrhosis death cases per 100,000 female population aged 15 or olderyear		alcl		cig		lcdcm	lcdcf		lcmom	lcmof1952		5.48		1,509	3,072	1,727	16.80	7.891953		5.80		1,553	3,633	2,051	19.50	9.231954		6.01		1,577	4,008	2,216	21.13	9.821955		6.68		1,661	4,376	2,348	22.60	10.251956		7.30		1,741	4,803	2,736	24.88	11.891957		7.91		1,822	5,260	2,952	26.98	12.701958		8.20		1,874	5,475	3,070	27.72	13.071959		8.95		1,953	6,184	3,505	31.02	14.811960		9.70		2,053	6,688	3,824	33.20	16.101961		10.75	2,174	6,877	3,849	33.86	16.081962		11.40	2,253	7,294	4,008	35.49	16.661963		12.06	2,272	7,687	4,229	37.07	17.461964		12.33	2,380	7,686	4,392	36.57	18.001965		13.13	2,455	8,347	4,886	39.20	19.881966		13.04	2,541	8,446	5,095	39.49	20.611967		12.99	2,497	8,557	4,946	40.15	19.981968		13.59	2,609	9,192	5,357	42.74	21.531969		14.25	2,703	9,477	5,358	43.26	21.361970		14.95	2,810	9,684	5,182	44.34	20.681971		15.84	2,919	9,894	5,297	44.69	20.971972		15.52	2,905	10,180	5,359	45.51	21.041973		15.92	2,862	10,643	5,265	46.97	20.501974		15.21	2,899	11,194	5,497	49.33	21.301975		16.16	2,815	11,650	5,598	51.37	21.631976		16.62	2,916	11,715	5,565	51.43	21.411977		15.84	2,715	11,341	5,581	49.40	21.331978		15.69	2,820	11,343	5,596	48.93	21.241979		15.93	2,825	10,223	5,157	43.50	19.401980		15.62	2,863	10,029	5,005	42.06	18.671981		15.15	2,875	10,131	5,132	42.05	19.001982		14.85	2,691	9,358	4,872	38.64	17.941983		14.88	2,745	9,377	4,746	38.55	17.421984		14.32	2,713	8,800	4,633	36.06	16.971985		14.30	2,711	8,714	4,751	35.53	17.361986		13.91	2,646	8,151	4,377	33.03	15.951987		14.00	2,644	8,289	4,633	33.38	16.871988		13.81	2,597	8,466	4,695	33.85	17.021989		13.64	2,601	8,722	4,890	34.35	17.561990		13.76	2,531	8,450	4,869	32.72	17.321991		13.88	2,466	11,745	6,226	36.57	17.761992		13.78	2,281	11,735	6,132	36.10	17.411993		13.47	2,193	12,095	6,290	37.00	17.801994		13.35	2,252	12,165	6,240	37.06	17.621995		13.32	2,244	11,972	6,033	36.24	16.971996		13.09	2,236	11,871	5,870	35.77	16.471997		12.99	2,263	11,499	5,661	34.56	15.861998		12.73	2,290	11,113	5,516	33.33	15.441999		12.76	2,380	10,848	5,560	32.41	15.532000		12.61	2,306	11,001	5,320	32.74	14.832001		12.44	2,360	10,710	5,407	31.69	15.012002		12.21	2,390	10,900	5,350	32.08	14.802003		11.89	2,250	10,988	5,244	32.21	14.472004		11.82	2,043	10,338	4,865	30.20	13.392005		11.65	1,948	10,081	4,762	29.34	13.072006		11.91	1,809	9,698	4,719	28.17	12.952007		11.60	1,812	9,320	4,369	27.03	11.992008		11.49	1,717	9,249	4,566	26.83	12.552009		11.21	1,687	9,131	4,419	26.51	12.162010		11.06	1,663	9,129	4,518	26.46	12.442011		11.31	1,782	8,973	4,449	26.62	12.412012		11.21	1,689	8,978	4,270	26.48	11.882013		11.13	1,630	8,940	4,449	26.22	12.35
