# Supplementary material for: Liver cirrhosis mortality, alcohol consumption and tobacco consumption over a 62 year period in a high alcohol consumption country: a trend analysis
Source: BMC Res Notes. 2015 Dec 26;8:822. doi: 10.1186/s13104-015-1808-2 (PMC4691532; doi:10.1186/s13104-015-1808-2)
Supplement: Supplementary file 2 — 10.1186/s13104-015-1808-2 Data file 2 (substance use disorder treatments). [file 13104_2015_1808_MOESM2_ESM.rtf]

Data file 2SUD		Substance use disorder treatments per 100,000 		population aged 15 or olderyear		SUD1982 	32.731983 	36.101984 	42.741985 	44.541986 	43.411987 	45.631988 	46.431989 	46.451990 	45.881991 	40.141992 	42.941993 	44.831994 	47.791995 	49.621996 	50.981997 	60.131998 	61.801999 	63.222000 	66.942001 	70.092002 	73.852003 	72.652004 	74.442005 	71.812006 	69.882007 	79.502008 	78.972009 	81.182010 	80.492011 	77.592012 	77.522013 	73.03
