# Supplementary material for: Liver cirrhosis mortality, alcohol consumption and tobacco consumption over a 62 year period in a high alcohol consumption country: a trend analysis
Source: BMC Res Notes. 2015 Dec 26;8:822. doi: 10.1186/s13104-015-1808-2 (PMC4691532; doi:10.1186/s13104-015-1808-2)
Supplement: Supplementary file 3 — 10.1186/s13104-015-1808-2 Data file 3 (hospital treatment cases). [file 13104_2015_1808_MOESM3_ESM.rtf]

Data file 3Hospital treatment cases, discharge diagnosis groupsk70m	ICD-10 diagnosis group K70, male inpatientsk70f		ICD-10 diagnosis group K70, female inpatientsk74m	ICD-10 diagnosis group K74, male inpatientsk74f		ICD-10 diagnosis group K74, female inpatientsk7074m	ICD-10 diagnosis group K70 or K74, male inpatientsk7074f	ICD-10 diagnosis group K70 or K74, female inpatientsyear		k70m	k70f		k74m	k74f		k7074m	k7074f2000		62.89	26.03	37.11	27.57	100.00	53.602001		62.54	25.78	38.05	28.43	100.59	54.212002		58.07	24.06	43.02	29.28	101.08	53.342003		62.83	25.54	39.57	28.00	102.40	53.542004		67.02	26.64	32.73	25.19	99.75	51.832005		73.19	28.03	34.27	26.41	107.45	54.452006		72.86	28.46	33.04	26.34	105.91	54.802007		72.21	29.21	31.19	25.06	103.40	54.272008		76.07	29.33	30.40	24.26	106.47	53.592009		77.56	30.57	30.24	23.86	107.80	54.432010		77.30	30.02	31.31	25.41	108.61	55.432011		80.18	30.32	32.20	25.92	112.38	56.242012		78.71	30.08	33.30	27.02	112.01	57.102013		78.66	29.92	34.05	27.34	112.71	57.25
